# Neurocognitive impairment in addiction: A digital tool for executive function assessment

**DOI:** 10.3389/fpsyt.2022.955277

**Published:** 2022-10-05

**Authors:** Michela Balconi, Doriana Losasso, Alessandra Balena, Davide Crivelli

**Affiliations:** ^1^International Research Center for Cognitive Applied Neuroscience (IrcCAN), Catholic University of the Sacred Heart, Milan, Italy; ^2^Research Unit in Affective and Social Neuroscience, Department of Psychology, Catholic University of the Sacred Heart, Milan, Italy; ^3^SerD Canzio, Department of Mental Health and Dependence, ASST Fatebenefratelli-Sacco, Milan, Italy

**Keywords:** addiction, executive functions, assessment, substance use disorder, cognitive screening

## Neurocognitive and EF impairment in psychopathology: A focus on addiction

Many models of Executive Functions (EF) can be found in literature, yet they are almost universally considered as core constituents of higher cognition. While extensive overlap between the conceptual definitions of EF and of the neighboring construct of metacognition has been critically noted [for a discussion on this topic, see (1)], EF are systematically described as a family of top-down, effortful processes that we rely on to effectively manage cognitive resources and direct behavior when a situation or task requires intentional, strategic, or planned responses, while automatic processing or learned basic responses would not be sufficient or advisable (2). Given their role in supporting self-monitoring, self-regulation, and top-down control of cognitive processes, EF represent a primary mediator of both typical and atypical functioning, with sometimes subtle influence on development and progress of psychopathology.

The transdiagnostic nature of altered EF and neurocognitive impairments in psychopathology is further suggested by the limited evidence for selective and specific cognitive symptoms between psychiatric disorders, an observation that contrasts with the vast amount of empirical observation concerning differences with respect to healthy control subjects. Deficits of EF, specifically, and neurocognitive impairments, globally, showed a systematic—though often underestimated—association with most psychopathological pictures (3–5) and might, therefore, be considered one of the most common transdiagnostic feature across the lifespan (6, 7).

Focusing on both substance use disorder and behavioral addictions, and going down to specifics with regard to cognitive alterations pairing with such psychiatric pictures, neurocognitive deficits especially affecting higher cognition have been widely documented by numerous studies (8–11). Specifically, executive impairments involving inhibitory control, attribution of salience to stimuli, decision-making, goal-oriented behavior, flexibility in selecting and initiating an action, inverted learning, and error tracking (11–13) have been reported and linked to alterations of the mesocorticolimbic dopaminergic circuits and of the corticostriatal glutamatergic circuits in prefrontal regions. Notably, according to neurocognitive models of addiction (13, 14), the negative impact of such cognitive deficits is further increased since they contribute to making more difficult to decide to stop using the substance of abuse or enacting dysfunctional behaviors, as well as to persist in this decision (15, 16). The ability to develop conscious decision-making strategies and the efficiency of self-awareness and metacognition also appear to be partially compromised, as happens in pathologies that involve similar deficits in neural circuits that foster decision-making processes (17–19).

Besides metacognitive and emotion regulation skills, the cognitive domains that most consistently showed the greatest vulnerability in individuals who developed substance and/or behavioral addiction are: attention regulation, learning and memory, inhibitory control, working memory, decision-making, cognitive flexibility, and strategic orientation of cognitive resources (11, 20). Yet again, on top of generalized impairments transversal to different addiction pictures, specific cognitive deficits depending on the substance of abuse and other individual factors such as the duration of abstinence have also been reported, though a certain degree of inconsistency in such empirical observations has to be acknowledged (9). Namely, the most consolidated data suggest that persistent use of psychostimulants (e.g., MDMA and cocaine) specifically affects inhibitory control and impulsivity, cognitive flexibility, working memory, as well as emotional regulation. Repeated use of opioids, instead, primarily hinders decision-making and the efficiency of regulation and distribution of attention resources, working memory, and cognitive flexibility. Moving to preliminary findings coming from clinical research on behavioral addictions (e.g., problematic internet use, gaming disorder, pathological gambling, compulsive buying disorder), the mostly observed cognitive impairments concern inhibition mechanisms, executive control skills (with implications on attention regulation, decision-making, and working memory), altered sensitivity (i.e., dysfunctional attribution of salience) to specific stimuli of interest, and impulse control (10, 11, 21, 22).

## Assessing EF in addiction

Following the premises above, it has now to be acknowledged that—notwithstanding the critical role of EF dysfunction in shaping clinical manifestations of substance-related and behavioral addiction—a complete definition of relationships and inter-dependences between models of abuse, addiction-related neurofunctional alterations, and specific patterns of neurocognitive/EF impairments still is a complex and almost unsolved issue. We here state that part of the reason of such open questions is the lack of dedicated assessment tools capable of detecting, qualifying, and quantifying the specific set of altered higher cognitive functions in patients who have developed addiction, taking into account its peculiar and often subtle manifestations.

Indeed, assessment practices at psychiatric emergency or addiction assistance/treatment services typically rely on basic aspecific screening batteries when the evaluation of cognitive and executive dysfunction is proposed. The short administration and correction times and fairly easy possibility to sketch the patient's global functioning profile that connote short cognitive batteries typically explain why they are commonly preferred to longer in-depth neuropsychological assessment batteries. Among the most used cognitive screening tools, those who are most represented in both clinical practice and among empirical investigations are the Mini Mental State Examination [MMSE (23)], the Neurobehavioral Cognitive Status Exam [NCSE (24)], the Brief Assessment of Cognition in Schizophrenia [BACS (25)], the Montreal Cognitive Assessment [MoCA (26)], the Neuropsychological Assessment Battery-Screening Module (NAB-SM; 27), the Addenbrooke's Cognitive Examination- Revised [ACE-R (27)], or the Frontal Assessment Battery (28–30). Notably, the MoCA (31, 32) and the NAB-SM (31, 33, 34) have been specifically tested as tools for cognitive screening in subjects with substance use disorder. Yet, while in the former the peculiar weight of memory and orientation subtests with respect to other cognitive domains (including EF) hinders the sensitivity of the MoCA in profiling executive deficits, the broader focus of the latter lacks of subtests dedicated to functions that are typically affected by addiction, such as inhibitory control, whereas providing pieces of information on cognitive functions that are not at the core of addiction-related neurocognitive impairments.

Overall, it should be remembered that such aspecific screening tools were originally devised to screen or assess cognitive impairment or dysexecutive syndrome in different clinical population than people presenting addiction, such as neurology or geriatric patients, or individuals with Multiple Sclerosis, schizophrenia, or neurodegenerative diseases. While such clinical cohorts might share some cognitive alterations with people presenting substance-related or behavioral addictions (e.g., dysfunctional regulation of attention resources, altered processing speed, and compromised inhibitory mechanisms), those tools might not be optimal to evaluate clinical populations different from the ones they were developed for, as shown in other clinical contexts (35, 36). People who have developed substance-related and, even more, behavioral addiction, are, indeed, typically younger than reference clinical cohorts used to validate, as an example, screening tools designed for geriatric patients, and might present—especially at the beginning of the history of substance use or implementation of dysfunctional behaviors—more subtle impairments, that require finer-grained evaluation (31, 32).

## Rethinking evaluation of addiction: A novel digitalized battery for neurocognitive assessment of EF

We propose that neurocognitive assessment of EF dysfunctions associated with addiction should represent a crucial—as well as currently underrepresented—step of the diagnostic process in routine assessment practice at drug assistance/treatment services. Namely, being able to point out cognitive vulnerabilities and to discriminate the nature of EF deficits in the earliest stages of the clinical history would complement clinical interviews with patients and their relatives and help designing the therapeutic plan, possibly planning a parallel cognitive rehabilitation phase. Such an approach might also indirectly impact on compliance and perceived efficacy of interventions, in that the impairment of executive functions likely hinders the acceptance and commitment in recovery programs and patient's sense of efficacy in the activities of daily life.

In order to answer such clinical, methodological, and practical questions we have designed and implemented a novel digitalized assessment tool, devised to be modular, easy, and relatively brief in its administration. The tool—named Battery for Executive Functions in Addiction (BFE-A)—consists of seven subtests (see Table 1) and includes measures dedicated to short- and long-term verbal memory, working memory, cognitive flexibility investigated with both verbal and non-verbal materials, focused attention, attention regulation and suppression of interference and inhibitory control. The set of tests and tasks that constitutes the BFE-A was selected based on their relevance, as highlighted by empirical literature, and their diagnostic potential, as highlighted by available psychometric and clinical evidence [further information on the selection and revisions of subtests included in the finalized version of the tool, as well as on technical specifications on the tool development process, can be found in (37)].

**Table 1:** Structure of BFE-A subtests, cognitive functions and processes investigated by each subtest, and their type.

**Subtest**	**Primary cognitive function**	**Specific processes**	**Type**
Verbal Memory Test—VMT	Short/long-term memory	Encoding consolidation, and retrieval of verbal material presented in auditory mode	Digitalized test
Working Memory Test—WMT	Working memory	Transient storage and active processing of numerical material presented in auditory mode	Digitalized test
Focused Attention Test—FAT	Focused Attention	Detection and selection of target visual-spatial stimuli, inhibition of distracters	Digitalized test
Verbal Fluency Test—VFT	Cognitive flexibility	Lexical access and selection, self-monitoring, set shifting, with linguistic material	Digitalized test
Non-verbal Fluency Test—NvFT	Cognitive flexibility	Generative and creative processes, self-monitoring, set shifting, with visual spatial patterns	Digitalized test
Modified Stroop Task for Addiction—MST	Attention regulation and suppression of interference	Control of interference due to semantic incongruence or salience of addiction-related stimuli	Computerized task
Modified Go/No-go Task for Addiction—MGNT	Inhibitory control	Suppression of pre-potent responses, control of attention bias for addiction-related stimuli	Computerized task

Besides outlining a general profile of preserved and compromised EF and higher cognitive processes associated with addiction pictures, the battery subtests have been designed to allow calculation of specific performance indicators for each cognitive domain, as well as inter-test comparison of performance. Also, each subtest has been complemented with different performance indices and error indices, devised to be metrically comparable. That allows the examiner to run intra-individual inter-test comparisons of performance at the different investigated functions, as well as to draw parallels between the examinee's performances at the various subtests. Such form of profiling, by pointing out both strengths and weaknesses of the examinee, would make easier to take into account patient's potential and specific needs when planning targeted diagnostic investigations or personalized empowerment/rehabilitation interventions. In addition, the comparability of subtest outcomes allows identifying specific effects of an implemented treatment protocol by weighing them transversely to the investigated cognitive domains. Such features provide valuable hints for the optimization or efficiency testing of different care and assistance plans.

Furthermore, in order to try and account for the second practical and methodological need for a flexible and properly informative test, the battery has been fully implemented online, it being constituted by digitized neuropsychological tests and computerized neurocognitive tasks. Computer-based performance testing, indeed, allows for a remarkable level of control over the procedures of test administration and high precision in the presentation of stimuli and collection of responses. Such greater sensitivity and discriminating capacity even in case of milder impairment becomes peculiarly relevant when applied to screen attention regulation skills and the efficiency of interference inhibition and cognitive control mechanisms. Indeed, the consequences even of minor alterations of those essential executive skills may affect behavior and everyday life while being hidden by compensation mechanisms that make them more difficult to identify via traditional aspecific paper-and-pencil tests.

To sum up, four methodological principles have guided the design of the battery:

- *Psychodiagnostic value and clinical relevance*: optimal coverage of and opportunity to explore executive deficits primarily associated with substance-related and behavioral addiction.
- *Modularity*: possibility of using the subtests of the battery also as independent tests or of creating subsets of tests for specific diagnostic investigations, in addition to the use of the battery as a unitary tool for screening the executive functioning of the examinee. Such a modular structure also provides professionals with the opportunity to adapt assessment to patients' attention capacities or time limitations.
- *High clinical informativity*: ability to provide an overall profile of integrity of the examinee's EF and higher cognitive skills that could then possibly be complemented by second-level neuropsychological assessment. Such an approach would optimize the resources dedicated to assessment procedures.
- *Clinical usability*: rapid administration and correction times, as well as selection of materials and methods of administration that could be easily implemented and are simple to use in real-life clinical settings, regardless of specific technological equipment and digital facilities available at the service.

## Conclusion

According to a neurodevelopmental model of addiction, many factors and life events may shape the relationship between executive impairments and psychopathology. Yet, despite the relevance of neurocognitive skills, no proper nosology of executive and higher-cognition deficits in psychopathology have been developed and intervention protocols that specifically target them are very scant and understudied. A unified model for classifying and recognizing neurocognitive impairments in psychopathology, and specifically addiction, is needed, as well as validated tools to assess the extent and severity of such impairments in specific clinical populations.

Also, taking into account the boundaries of the setting that connote assessment practices at drug assistance/treatment services, we propose that the use of a cognitive screening battery created *ad hoc* for the target clinical population—possibly followed, if needed, by second-level diagnostic investigations—constitute a good compromise between the accuracy of a complete evaluation and the specificity of an assessment that is completely tailored on the individual but may require remarkable clinical experience and time to be properly set-up.

## Author contributions

DC wrote the first draft of the manuscript. AB, DL, and MB revised it. All authors contributed to conception of the present work and read and approved the submitted version.

## Conflict of interest

The authors of this manuscript also are the developers of the Battery for Executive Functions in Addiction [Batteria per le Funzioni Esecutive nell'Addiction–BFE-A], an assessment tool that was born from the clinical and methodological need for population-specific tests of higher cognitive functioning in addiction.

## Publisher's note

All claims expressed in this article are solely those of the authors and do not necessarily represent those of their affiliated organizations, or those of the publisher, the editors and the reviewers. Any product that may be evaluated in this article, or claim that may be made by its manufacturer, is not guaranteed or endorsed by the publisher.
